# Ocular Metastasis in Elderly Male Bladder Cancer Patients: Potential Risk Factors

**DOI:** 10.1177/1557988320908998

**Published:** 2020-03-07

**Authors:** Qian-Hui Xu, Qing Yuan, Yu-Qing Zhang, Biao Li, You-Lan Min, Qian-Min Ge, Rong-Bin Liang, Yi Shao

**Affiliations:** 1 Department of Ophthalmology, The First Affiliated Hospital of Nanchang University, Jiangxi Province Ocular Disease Clinical Research Center, Nanchang, Jiangxi, People’s Republic of China; 2 Department of Ophthalmology, The First Affiliated Hospital of Nanchang University, Jiangxi Province Clinical Ophthalmology Institute, Nanchang, Jiangxi, People’s Republic of China

**Keywords:** Elderly male, ocular metastasis, bladder cancer, alkaline phosphatase, hemoglobin, risk factors

## Abstract

Bladder cancer is a common type of tumor among elderly male population; it causes intraocular metastasis (IOM). The study investigated the differences between elderly male bladder cancer patients with and without IOM, and identified risk factors for IOM. In this study, 749 elderly male patients (aged ≥50 years) with bladder cancer were included from November 2003 to December 2016. Differences between the IOM and non-IOM (NIOM) groups were evaluated by chi-square test and Student’s *t*-test. The binary logistic regression analysis calculates the risk factors. Receiver operating characteristic (ROC) curve analysis was used to assess the diagnostic value of IOM in elderly male patients with bladder cancer.

The incidence of IOM in patients with bladder cancer was 1.7%. No significant differences were detected in age and histopathology between the IOM and NIOM groups. According to the study, the IOM group had higher ALP and Cyfra21-1. Binary logistic regression indicated that ALP and Cyfra21-1 were risk factors for IOM in elderly male bladder cancer patients (*p* < .05). ROC curve analysis revealed area under the curve values for ALP and Cyfra21-1 of 0.913 and 0.814, using cutoff values of 9.65 and 83.5 U/L, respectively. The sensitivity and specificity values for ALP were 61.5% and 95.8%, respectively, while those for Cyfra21-1 were 84.6% and 73.3%.

The investigation indicates that ALP and Cyfra21-1 are risk factors for IOM in elderly male patients with bladder cancer and ALP is more reliable at distinguishing IOM from NIOM in elderly male patients with bladder cancer.

Cancer has become a disease associated with high morbidity and mortality (Ma & Yu, 2006). Bladder cancer is the most common malignant tumor of the urinary system, mostly occurring in middle-aged and elderly individuals. It ranks fourth among the different kinds of cancers and the incidence of bladder cancer is higher in male than female; at the same time, the incidence of bladder cancer is also increasing (Dal Moro et al., 2013). Besides prostate cancer, it can be identified that elderly males have high incidence of bladder cancer (Han et al., 2013).

The pathogenesis, diagnosis, and treatment of bladder cancer are of great importance. The clinical treatment of bladder cancer involves the use of surgery, chemotherapy, radiotherapy, and immunotherapy. A large number of patients experience recurrence and metastasis after treatment. Several studies have reported that metastasis of bladder cancer primarily occurs in the lymph nodes and lungs. Notably, metastases to the bones have also been reported (Bianchi et al., 2014). In addition, in several cases, metastasis of bladder cancer may occur in other sites. The limited number of studies regarding intraocular metastasis (IOM) of bladder cancer may be attributed to the low number of bladder cancer patients with IOM. Several investigations have reported that bladder cancer has become one of the risk causes of IOM (Giordano et al., 2019). In the setting of metastatic bladder cancer, distant lymph nodes and lung are the most common sites of metastases. Although IOM of bladder cancer is rare, some research indicates that the incidence of this condition is increasing. Studies have identified that IOM of bladder cancer can lead to ocular lesions, such as decreased vision, decreased ocular motility, double vision, and ocular herniation (Sklar et al., 2018). The identification of potential risk factors for IOM of bladder cancer has become particularly important to avoid the occurrence of such metastases.

Cystoscopy, radiography, and liquid cytology are the most important diagnostic methods for bladder cancer in clinical practice (Saeb-Parsy et al., 2012). The use of cystoscopy for the examination of patients with bladder lesions can clearly reflect the lesions in the bladder wall. However, it cannot accurately determine the degree of lesion invasion in the tissues and organs outside the bladder wall. This limitation may lead to misdiagnosis of the disease (Sullivan et al., 2009). Although urinary cytology is a noninvasive diagnostic method (Whisnant et al., 2003), this approach is characterized by lower sensitivity (Soyuer et al., 2009). The usefulness of these methods for the diagnosis of bladder cancer is limited. Consequently, our study finds that serological examination is a method of tumor diagnosis through the examination of biomarkers in the serum. Clinically, after testing cytokeratin fragment 19 antigen 21-1 (Cyfra 21-1), neuron-specific enolase (NSE), alpha fetoprotein (AFP), carcino-embryonic antigen (CEA), carbohydrate antigen 125 (CA125), CA153, CA199, CA724, alkaline phosphatase (ALP), hemoglobin (Hb), and calcium in the serum during the serological examination to find different values of serum markers between IOM and NIOM. According to some recent research and clinical usage, CA153 was highly expressed in a variety of epithelial adenocarcinoma cells and exhibited a high positive rate in serous and mucous ovarian cancer. AFP is the most sensitive and specific marker for the early diagnosis of primary liver cancer (Tsuchiya et al., 2015). CA125 is a glycosylated antigen that can be expressed in a variety of mesothelial tissues, and is highly expressed in ovarian cancer (Sasaki et al., 2015). CA199 is an oligosaccharide antigen, which is often used in the diagnosis of tumors of the digestive tract. CEA is also a specific glycogen antigen, and one of the serological markers of malignant tumors often expressed in epithelial tissues. Its combined application with other tumor markers in the serum is of great importance in the diagnosis of malignant tumors (Wang and Tian, 2014).

Serum biomarkers can indicate whether the tumor has metastasized to other tissues. The purpose of this study was to screen biomarkers that may guide the prediction of IOM of bladder cancer, by detecting biomarkers in the serum of elderly male patients with or without IOM of bladder cancer. In addition, the results were analyzed to identify potential risk factors for the occurrence of IOM of bladder cancer in elderly males. The study attempted to determine the correlation between various risk factors and IOM, and establish an accurate indicator to distinguish IOM from nonintraocular metastasis (NIOM) in this subset of patients. The goal is to lay the foundation for targeted local and systemic anticancer treatment strategies (Westerwick et al., 2017).

## Materials and Methods

### Ethics Statement

This study was approved by the Medical Research Ethics Committee of the First Affiliated Hospital of Nanchang University. Written informed consent was provided by all participants. All methods were performed in accordance with the tenets of the Declaration of Helsinki.

### Study Design

The study recruited 749 elderly male patients (aged ≥50 years) who were diagnosed with bladder cancer in the Department of Urology, Nanchang University First Affiliated Hospital, from November 2003 to September 2016. In the study, all the patients were divided into two groups: the bladder cancer-induced IOM group and the NIOM group. Patients with primary ocular malignancies, benign ocular tumors, and secondary bladder cancer were excluded from the study.

The inclusion criteria for the IOM group were as follows: bladder cancer patients without primary ocular malignancies, and benign ocular tumors (e.g., orbital inflammatory pseudotumors), but with IOM. The inclusion criteria for the NIOM component group were as follows: bladder cancer patients without organ metastasis, lymph node metastasis, and IOM. The medical records were constantly updated throughout the study. The diagnosis of bladder cancer was achieved through pathological examination. Moreover, IOM and other types of metastasis were diagnosed using computed tomography (CT) and magnetic resonance imaging. The patients did not receive relevant antitumor treatment. Various data such as pathological, lymph node metastasis, and bone metastasis were also collected. The expression levels of relevant tumor biomarkers were recorded through serological examination.

### Data Collection

All data analyzed in this study, including patient age, gender, primary cancer diagnosis, pathological type, presence or absence of metastasis, bone metastasis, lymph node metastasis, CT, and magnetic resonance imaging data, were obtained from medical records. Collection of all relevant data and collection and testing of blood samples were performed when the patient was initially diagnosed with bladder cancer. The concentration of Hb, ALP, and calcium as well as the expression levels of various tumor biomarkers (i.e., Cyfra21-1, NSE, AFP, CEA, CA125, CA153, CA199, and CA724) were determined in serum samples obtained from patients with bladder cancer. This study calculated the incidence of IOM in patients with bladder cancer, analyzed its clinical characteristics, and evaluated the possible relationships between IOM and related biomarkers in bladder cancer.

### Statistical Analyses

Chi-square test and Student’s *t*-test were used to assess the clinical features and differences between the bladder cancer IOM and NIOM groups. Independent risk factors for bladder cancer IOM were studied using a binary logistic regression model. Accuracy of the predictive diagnosis of IOM in elderly male patients with bladder cancer was assessed by plotting the receiver operating characteristic (ROC) curve and calculating the area under the curve (AUC). *p* < .05 denoted statistical significance. Statistical tests were performed using Microsoft Office Excel 2016 ((Excel, Microsoft Corporation, Redmond, WA, USA Microsoft Corp, USA), SPSS version 17.0 software (SPSS Inc., Chicago, IL, USA), and MedCalc 18.6.0 statistical software (MedCalc, Ostend, Belgium). Continuous data are expressed as mean ± standard deviation.

## Results

### Participants and Clinical Characteristics

**Figure 1.**
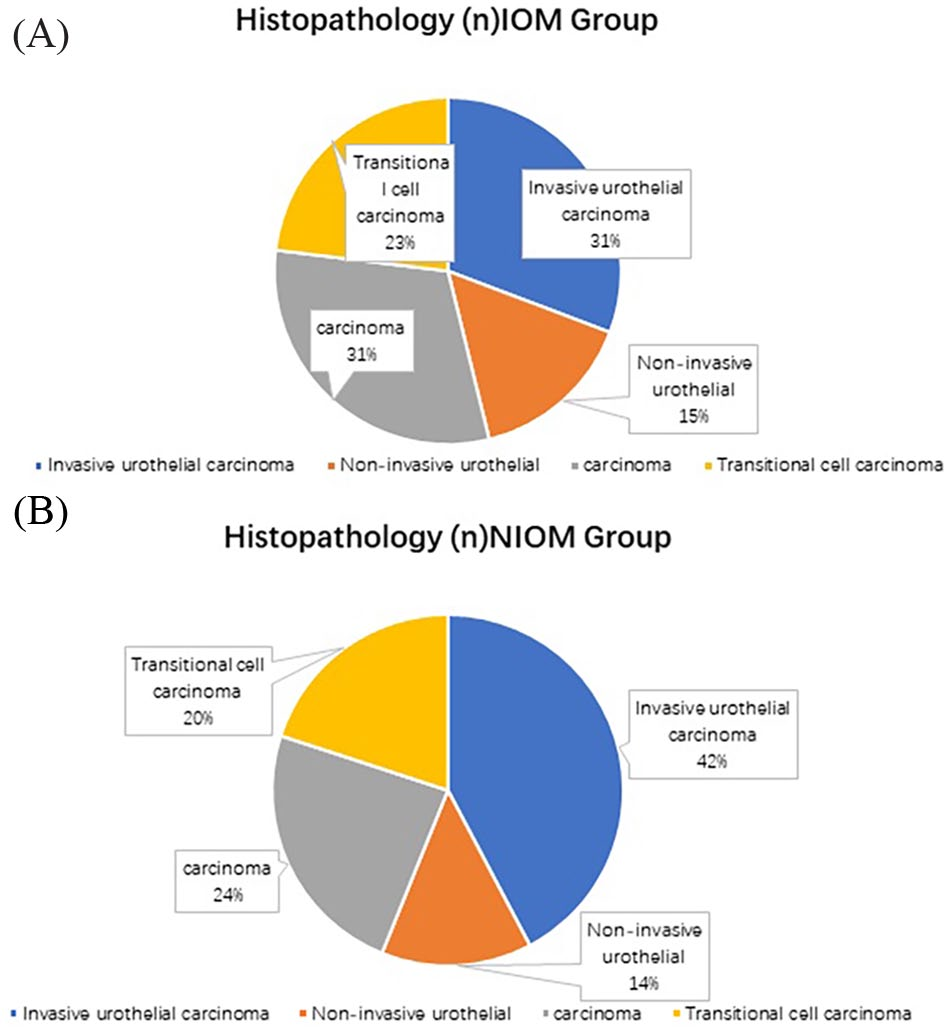
Clinical characteristics of bladder cancer patients with (A) IOM and (B) NIOM. *Note.* IOM = intraocular metastases; NIOM = nonintraocular metastases.

A total of 749 elderly male patients with bladder cancer participated in the study. Of these, 13 patients experienced IOM. The average age of bladder cancer patients with and without IOM was 67 and 65years, respectively. The types of carcinoma detected among the patients with IOM of bladder cancer were as follows: invasive urothelial carcinoma (four cases), noninvasive urothelial carcinoma (two cases), transitional cell carcinoma (four cases), mucinous adenocarcinoma (two cases), and other types (one case). The types detected among patients with NIOM were: bladder cancer-type invasive epithelial carcinoma (311 cases), noninvasive epithelial cancer (102 cases), transplanted cell carcinoma (176 cases), and other types of cancer (147 cases; Table 1 and Figure 1).

**Table 1.** Baseline Characteristics of Patients with Bladder Cancer.

Items	IOM group (%) (***n*** = 13)	NIOM group (%) (***n*** = 13)	Total number of patients (%)	***p*** value
**Age (years)**	67.15 ± 11.45	65.02 ± 11.33	65.05 ± 11.33	
**Histopathology (*n*)**				
**Invasive urothelial carcinoma**	4 (30.8)	311 (42.3)	315 (42.1)	.0069
**Noninvasive urothelial carcinoma**	2 (15.4)	102 (13.9)	104 (13.9)	
**Transitional cell carcinoma**	4 (30.38)	176 (23.9)	180 (24.0)	
**Other types**	3 (23.1)	147 (19.9)	149 (19.9)	

*Note.* IOM = intraocular metastases; NIOM = nonintraocular metastases.

Student’s *t*-test and chi-square test were used. *p* < .05 represented statistical significance.

### Risk Factors for IOM in Patients With Bladder Cancer

In this study, the levels of ALP, Hb, and so forth in blood samples obtained from patients with bladder cancer have been tested. Results showed that the average level of ALP in patients with IOM and NIOM was 314.38 ± 717.68 U/L and 89.68 ± 125.07 U/L, respectively. In addition, the data showed that the level of Cyfra 21-1 in the blood of patients with IOM and NIOM was 50.84 ± 118.89 g/L and 9.58 ± 3.99 g/L, respectively. Notably, both differences were statistically significant (*p* < .05). The comparison did not show a significant difference in the concentration of calcium, CA724, and so forth in the blood of IOM and NIOM patients (*p* > .05). The results of the binary logistic regression model showed that the levels of ALP and Cyfra21-1 could be used as independent risk factors for IOM (Table 2).

**Table 2.** Differences in the Clinical Lipid-Relevant Parameters Between Patients With and Without IOM (Mann–Whitney test) and Results of the Binary Logistic Regression Analysis.

Factors	IOM group	NIOM group	***B***	OR	OR (95% CI)	***p***
ALP (U/L)*	314.38 ± 390.45	81.96 ± 78.16	0.0001	1.005	[1.001, 1.010]	.019
Cyfra21-1 (ng/ml)*	50.84 ± 132.41	4.75 ± 2.37	0. 378	1.460	[1.237, 1.723]	<.001
Serum calcium	2.37 ± 0.23	2.24 ± 0.24	2.160	8.673	[0.423, 177.623]	.161
CA-724 (U/ml)	42.02 ± 91.55	35.60 ± 32.93	0.007	1.007	[0.997, 1.017]	.152
NSE (μg/L)	48.65 ± 36.52	40.89 ± 18.50	−0.003	0.997	[0.985, 1.038]	.890
AFP (ng/ml)	3.45 ± 1.86	2.85 ± 1.17	0.086	1.090	[0.661, 1.799]	.735
CEA (ng/ml)	18.45 ± 39.78	15.83 ± 16.23	−0.055	0.947	[0.896, 1.000]	.051
CA-125 (U/ml)	363.54 ± 407.77	205.85 ± 239.71	0.000	1.000	[0.999, 1.002]	.476
CA-153 (U/ml)	83.24 ± 85.26	151.69 ± 88.68	−0.007	0.993	[0.982, 1.004]	.205
CA-199 (U/ml)	126.36 ± 271.81	106.83 ± 135.10	−0.006	0.994	[0.983, 1.005]	.279
Hb (g/L)	101.15 ± 20.92	117.02 ± 24.79	−0.023	0.997	[0.945, 1.010]	.177

*Note.* ALP = alkaline phosphatase; IOM = intraocular metastases; NIOM = nonintraocular metastases; Hb = hemoglobin; *B* = coefficient of regression; OR = odds ratio; CI = confidence interval; IOM = intraocular metastases.

Independent samples *t* test was applied. Binary logistic analysis was applied. *p* < .05 represented statistical significance.

### Diagnostic Value of ALP and Cyfra 21-1 in IOM of Bladder Cancer

**Figure 2.**
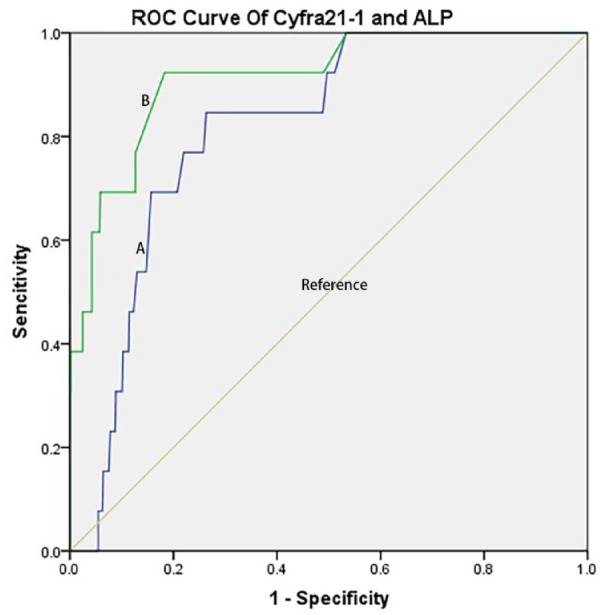
The ROC of ALP and Cyfra21-1 in bladder cancer patients with IOM and NIOM. *Note.* (A) ROC curve of Cyfra21-1. AUC was 0.814 (*p* < .001; 95% CI [1.237, 1.723]) (IOM > NIOMs); (B) ROC curve of ALP. AUC was 0.913 (*p* = .019; 95% CI [1.001, 1.010]) (IOM > NIOM). ROC = receiver operating characteristic; AUC = areas under the curve; CI = confidence interval; IOM = intraocular metastases; NIOM = nonintraocular metastases.

In this study, the ROC curves of ALP and Cyfra 21-1 have been mapped in order to assess the diagnostic value of ALP and Cyfra 21-1 for IOM of bladder cancer in Figure 2, and the Cutoff value, sensitivity, specificity and AUC values are shown in Table 3.

**Table 3.** Cutoff value, Sensitivity, Specificity, and AUC for Each Risk Factor in Predicting IOM in Patients With Bladder Cancer.

Factors	Cutoff value	Sensitivity (%)	Specificity (%)	AUC	***p***
ALP	9.65	61.5	95.8	0.913	.019
Cyfra21-1	83.5	84.6	73.3	0.814	<.001

*Note.* ALP = alkaline phosphatase; AUC = area under the curve; IOM = intraocular metastases; ROC = receiver operating characteristics.

## Discussion

Bladder cancer is the most common malignancy of the urinary system and the ninth most common cancer worldwide. There are approximately 400,000 newly diagnosed cases annually, and this number is increasing (Yaxley, 2016). According to the level of tumor infiltration in the bladder wall, bladder cancer can be divided into the muscularis invasive bladder cancer (i.e., nonmuscle invasive bladder cancer) and muscle layer of invasive bladder cancer (i.e., muscle-invasive bladder cancer). If patients can get accurate diagnosis and effective treatment at an earlier time, the 5-year survival rate of patients with bladder cancer can reach 95.7% (David et al., 2009). Following the occurrence of metastasis, the survival rate is only 5%. A large number of studies have identified the risk factors for lymph node and bone metastases of bladder cancer (Table 4). Research regarding IOM of bladder cancer has not been performed. The present analysis and comparison of clinical data showed that IOM also occurred in patients with bladder cancer. The incidence of IOM ranges from 2.5% to 8.1% (Katsourakis et al., 2014). The pattern of tumor metastasis varies between individuals; however, it is generally associated with poor prognosis.

**Table 4.** Risk Factors for Metastasis of Bladder Cancer.

Author (year)	Metastatic sites	Risk factor
Keck et al. (2013)	lymph node	SNAl1
Seiler et al. (2014)	lymph node	CyclinD1
Kawahara et al. (2016)	lymph node	NLR
Huang et al. (2017)	bone	ALP, Hb, Calcium
Xu et al. (2018)	lymph node	Arp2

*Note.* IOM = intraocular metastases.

The IOM of tumors have been reported in a variety of cancers. Most cases of IOM are adenocarcinomas, and the most common primary site is breast cancer (Whisnant et al., 2003). In general, different metastatic sites can lead to varied clinical manifestations. There are no clear diagnostic criteria, owing to the low incidence of IOM. The IOM of bladder cancer cannot be determined through clinical follow-up and CT examination, and is usually not detected at an early stage. Its diagnosis has been challenging, resulting in poor prognosis among patients diagnosed with late-stage metastasis. The assessment of tumor fluid has attracted considerable attention, and is expected to become the main screening method for the early screening of tumors.

In this study, elderly males with bladder cancer were analyzed, and the clinical data of patients with and without IOM were compared. The discovery of ALP and Cyfra21-1 as potential biomarkers can assist physicians in the early diagnosis of IOM of bladder cancer in these patients.

The prognosis of tumors is related to the biological characteristics, differentiation, and staging (Wen et al., 2017). Studies have suggested that the level of ALP in the serum may be an effective indicator of tumor proliferation and progression in liver cancer, esophageal cancer, nasopharyngeal cancer, and so forth (Yang et al., 2015). ALP has the potential to become a biological predictor of the prognosis of tumors, and may influence the long-term prognosis. Studies have reported that ALP is a tumor-related antigen. In addition, it has been identified that a high level of ALP promotes tumor cells to obtain stem cells, consequently leading to poor prognosis (Rao et al., 2017). The heterotopic expression of ALP is closely related to various human cancers. The expression level of the ALP isozyme is altered in malignant tissues. The levels of ALP in the serum were significantly increased in patients with hepatocellular carcinoma or breast cancer (Kim et al., 2017; Xu et al., 2014). Studies investigating changes in the levels of ALP in IOM of bladder cancer are limited. This study found that the AUC value of ALP was 0.913. The optimal diagnostic value was 9.65 U/L. Data processing showed that the sensitivity and specificity of ALP were 61.5% and 95.8%, respectively. A level of ALP >9.65 U/L was linked to an increased risk of developing IOM of bladder cancer.

The present study found that change in the expression level of Cyfra21-1is also an important biological marker for the IOM of bladder cancer in elderly males. The Cyfra21-1is mainly expressed in epithelial cells. Following the progression of epithelial cells into cancerous cells, the expression of Cyfra21-1 is upregulated and the antigen is gradually released into the peripheral blood. Cyfra21-1 is a diagnostic marker for potential epithelial-derived tumors. There is a positive correlation between the levels of Cyfra21-1 in the serum and urine. This observation suggests that, during the development of bladder cancer, the highly expressed cytokeratin in tumor tissue can enter the blood, consequently increasing the level of Cyfra21-1 in the blood. This study found that the AUC value of Cyfra21-1was 0.814. The best diagnostic value of Cyfra21-1 was 83.5 U/L. The sensitivity and specificity of Cyfra21-1were 84.6% and 73.3%, respectively. A level of Cyfra21-1 >83.5 U/L is associated with an increased risk of developing IOM of bladder cancer.

The present study was characterized by some limitations. The number of elderly male patients with bladder cancer in the study was limited. The study may not be generalizable to the nationwide population of elderly male bladder cancer patients with IOM. At the same time, the study only analyzed blood samples obtained from patients after IOM, without performing clinical follow-up which may lead to some errors.

In conclusion, the research has found that the expression levels of ALP and Cyfra21-1 in the serum of elderly male bladder cancer patients with IOM were significantly higher than those reported in bladder cancer patients without IOM. Through a series of statistical analyses, ALP and Cyfra21-1 in elderly male patients with bladder cancer metastasis have statistically difference (*p* < .05). ALP and Cyfra 21-1 in the prediction of IOM in bladder cancer can be used as a diagnostic factor. Both can be used for early intervention and treatment of early diagnosis of bladder cancer in older males shifted in the eye. Early diagnosis is associated with better prognosis versus late diagnosis. An early good prognosis can effectively reduce the risk of tumor metastasis, and prevent visual impairment and eye injury. This may effectively improve the quality of life of elderly males with bladder cancer.
